# The influence of cold weather on the usage of emergency link calls: a case study in Hong Kong

**DOI:** 10.1186/s12911-015-0191-1

**Published:** 2015-08-13

**Authors:** Feng Chen, Paul SF Yip

**Affiliations:** Department of Statistics, The University of New South Wales, Sydney, Australia; Department of Social Work and Social Administration, The University of Hong Kong, Pokfulam Road, Hong Kong, China

**Keywords:** Auto-regression, Count data, Generalized linear auto-regressive moving-average (GLARMA) model, Generalized linear model (GLM), negative binomial, Nonlinear effect, Overdispersion, Poisson regression, Time series

## Abstract

**Background:**

In response to an unexpected long cold spell in February 1996 which killed more than 100 older adults (mostly living alone) in Hong Kong, the Hong Kong Senior Citizen Home Safety Association established a Personal Emergency Link Service to provide emergency contact to the older adults, which uses a telephone system to render emergency relief and total care service around the clock. To facilitate the dynamic and efficient allocation of service resources, it is crucial to understand the factors linked with use of the services and number of hospital admissions arising from PE link service.

**Methods:**

We initially use the Poisson generalized linear model (GLM) with polynomial effect functions of relevant covariates. If the time series of residuals from fitting the Poisson GLM reveals significant serial correlation, a Poisson generalized linear autoregressive moving average (GLARMA) model is refitted to the data to account for the auto-correlation among the time series of daily call numbers. If the data is overdispersed relative to the best fitting Poisson GLARMA model, then the negative binomial GLARMA model is refitted to account for any overdispersion. In all the models, dummy variables for weekdays and months are included to account for any cyclic trends due weekday effect or month of the year effect. The secular time trend is modeled by a polynomial function of calendar time over the study period. Finally any critical temperatures are identified by visually inspecting the graph of the effect function of temperature.

**Results:**

The weekday and month effects are both significant with Monday seeing more PE Link calls than Sunday and June seeing less than January. Temperature has significant effect on the PE Link call rate with the effect highly nonlinear. A critical temperature, below which excessive increase in PE link calls that lead to hospital admissions, is identified to be around 15 °C.

**Conclusion:**

Identifying a threshold temperature which generates an excessive increase in the expected number of PE Link calls would be useful in service provision planning and support for elderly in need of hospital admission.

## Background

In February 1996, more than 100 elderly people living alone in Hong Kong died during an unexpected long cold spell, which is excessive in comparison to the same period in the previous years. In response to this incident, in the same year a non-government organization, the Hong Kong Senior Citizen Home Safety Association (SCHSA), established a personal emergency (PE) link service to render emergency relief and total care service to all elderly and chronic invalids. All subscribers of this service are linked through an advanced communication system to a 24-hour call center. By pressing a button on a main unit or the button on a portable necklace type or wristwatch type remote trigger, the service user can establish communication to the call center through the main unit at home. As of November 2007 the accumulated number of users of this service has reached 100,000.

There is substantial variation in the daily numbers of calls received in the Center. To facilitate appropriate and efficient resource allocation it is desirable to be able to predict the intensity of service use based on factors that can be predicted with reasonable accuracy, such as weather conditions. To this end it is important to study the relation of the service use intensity and the weather conditions (for example, temperature and humidity) based on historical data. A recent work [1] about the association between the frequency of such PE Link calls and meteorological factors found that the effect of temperature on the call frequency was statistically significant and the effect function was roughly U-shaped. When the temperature reached about 30–32 °C, the health related PE Link call frequency started to increase. The major concern of the study was the negative impact of hot weather on health, and only used data in warm seasons in Hong Kong. A more recent study [2] on the association between hospital admissions and weather and other environmental variables in Hong Kong found increased admissions were linked to increases of temperature above a threshold during warm seasons and decreases of temperature below a threshold during cold seasons. Another study [3] also found strong associations between cold weather and mortality rate in Hong Kong and Taiwan during cold seasons. The main purpose of the current work is to examine the association between the frequency of PE Link calls that lead to hospital admissions and meteorological factors, especially cold weather, using year-around data regardless of the season.

## Data and methods of analysis

### Data

**Fig. 1:**
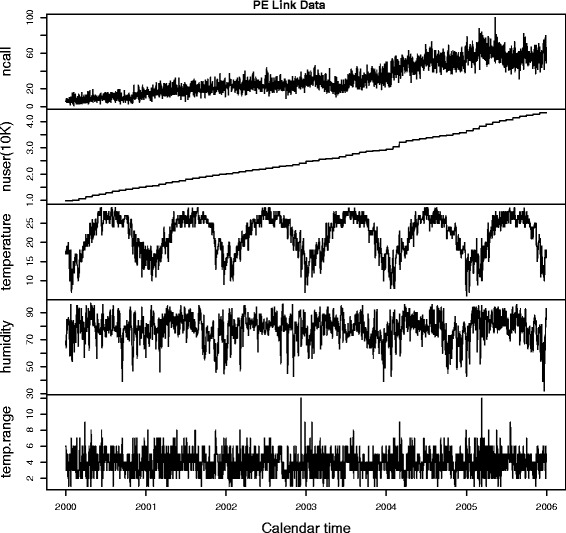
Time s eries plots of the data from 1 Jan 2000 — 31st Dec 2005. Top panel: daily number of PE Link calls that lead to hospital admission; Second panel: daily number of active subscribers (in 10,000) of the PE Link service; Third panel: daily minimum temperature (in degrees Celsius); Fourth panel: daily relative humidity (in percentages); Fifth and last panel: daily range of temperature, calculated as the difference between the daily maximum and minimum temperature values

The data supplied by SCHSA consist of the daily numbers of PE link calls that lead to hospital admission and effective service subscribers from 1 January 2000 to 31 December 2005. The daily weather information such as minimum temperature (in degrees Celsius) and relative humidity (in percentages) during this period, were downloaded from the Hong Kong Observatory’s historical weather database. See Fig. 1 for time series plots of the data.

### Methods

Since the daily numbers of calls are integer valued, it is natural to model them using Poisson distributions with parameters depending on explanatory variables. This motivated us to consider the Poisson GLM (Generalized Linear Model) regression as a first attempt. The response variable is the daily number of PE Link calls received by the call center that lead to hospital admissions. The explanatory variables included the daily temperature, the humidity and the range of temperature fluctuations. As our main concern is the impact of cold weather on service use, the temperature variable we use is the daily minimum temperature. The daily temperature range was calculated as the difference between the maximum and minimum recorded temperatures. Since previous studies suggest the effect of weather on health status tends to be nonlinear [1–6], the impact of each of these meteorological variables was modeled as a polynomial function over the respective range of the variable. The degrees of the polynomials were selected using a popular model selection method, the Akaike Information Criterion (AIC) [7].

To account for any potential seasonality effects associated with day of the week and month of the year, dummy variables for each of Monday, Tuesday,..., Saturday, and February, March,..., December were included in the explanatory variables, while Sunday and January were held as references. As the data plot shows non-cyclic variations in the daily call numbers, a polynomial function of calendar time over the study period was included to account for such secular trend. The degree of the polynomial for the time trend was selected together with the degrees of the polynomial effect functions for the meteorological variables in the model selection process using the AIC. As previous studies suggests that the severe acute respiratory syndrome (SARS) epidemic in 2003 might have influenced the call rate as well [8], the indicator for the duration of the SARS epidemic in Hong Kong, 4 Feb–21 May 2003, was also included in the model.

The terms in the best model at the end of the model selection process were each examined for statistical significance. Terms whose removal from the model would further reduce the AIC value were dropped to simplify the model. Due to the time series nature of the daily call counts, there might be auto-correlation among the call numbers, which, if left unaccounted for, could distort the inference on the covariate effects. Therefore, the residuals from fitting the simplified model was examined for significant auto-correlations by visually inspecting the ACF (auto-correlation function) plot. If significant auto-correlation was detected, then Poisson GLARMA (Generalized Linear Auto-Regressive Moving-Average) model [9, 10] would be fitted to the data to account for any auto-correlation among the count time series.

The residual deviance statistic after fitting the Poisson GLARMA model was checked against the residual degree of freedom of the model for evidence of overdispersion of the data relative the Poisson distribution. If overdispersion was detected, then a negative binomial GLARMA model would be fitted to the data to account for overdispersion and reduce the chance of falsely declaring significant covariate effects. Various diagnostic plots of the residuals from fitting the final Poisson or negative binomial GLARMA model were inspected to see if the model fits the data well. If the temperature variable was significant in the final model, then the estimated effect function for temperature would be graphed and visually inspected to determine any critical temperature.

## Results

The daily number of PE Link calls that lead to hospital admission ranges from 1–100, with mean 31.4, median 26, and SD (standard deviation) 18.3. The daily call rate per 10,000 subscribers ranges from 0.99–25.16, with mean 11.53, median 11.52, and SD 3.59. The daily minimum temperature ranges from 6–29 °C, with mean 21.8 °C, median 23, and SD 5.0. The daily humidity ranges from 32–97 %, with mean 78.2 %, median 79 and SD 9.7. The daily temperature range falls in the range 1–12, and has mean 3.93, median 4, and SD 1.4.

**Fig. 2:**
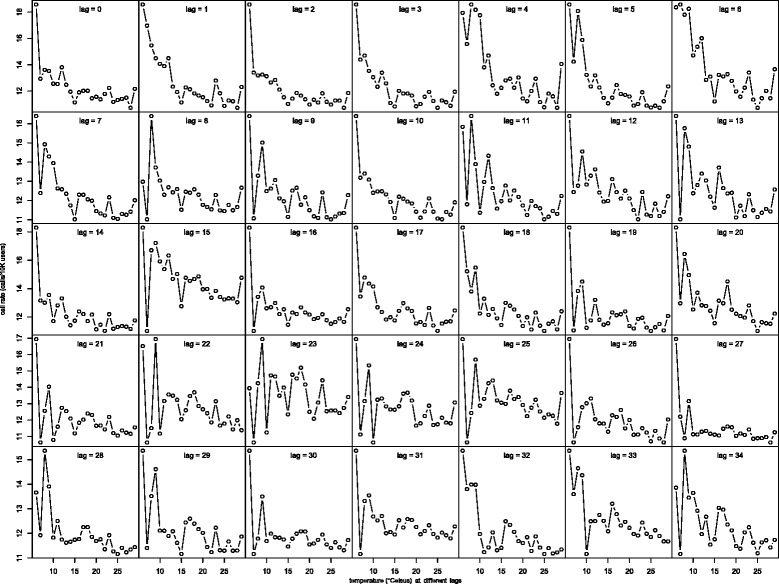
Smoothed PE Link call rate against temperature at different lags. Smoothed call rate at a specific temperature (or another variable) value was calculated by averaging all call rates associated with the specific temperature (or another variable) value

**Fig. 3:**
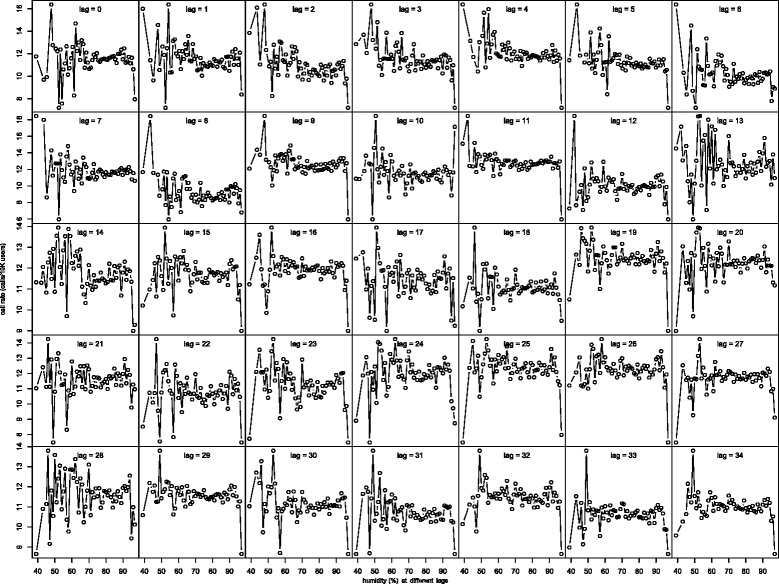
Smoothed call rate against humidity at different lags

**Fig. 4:**
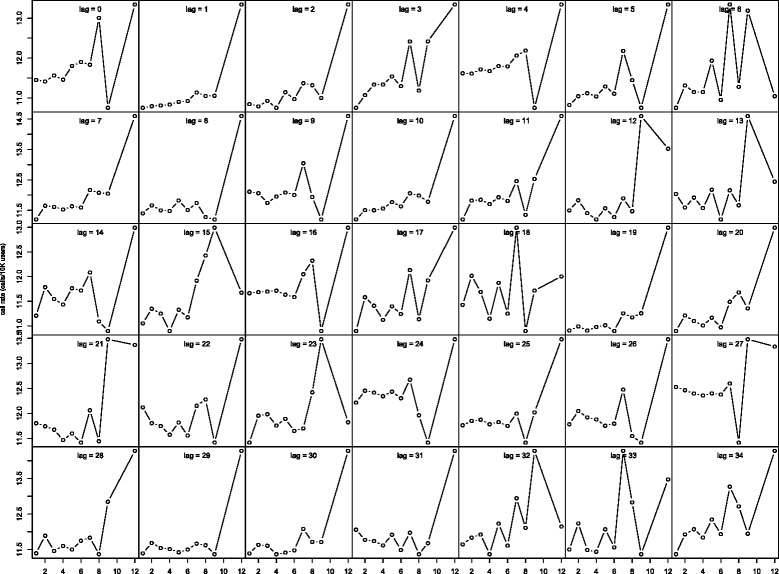
Smoothed call rate against temperature range at different lags

In Fig. 2, we graph the average call rate for each distinct temperature value as a function of temperature at different lags ranging from 0–34. The graphs shows somewhat similar relationships between call rate and temperature at different lags up to 34 days. This is not surprising as the time series of temperature shows strong auto-correlation that persists across different lags. In fact, the auto-correlation at lags 1–34 range from 0.952 to 0.643. Similar auto-correlation among the humidity and temperature range variables was also observed; see Figs. 3 and 4 for the graphs of the average call rate against humidity and temperature range respectively at different lags. In light of this issue of multicollinearity, we use the respective 7-day averages of the variable values on the same day and the previous 6 days as the corresponding meteorological variables in the subsequent regression analysis. We also tried to use averages of the variables up to 3 weeks, and the results were similar to those using the one-week averages to be reported below.

The degrees of the polynomial effect functions for temperature, humidity, temperature range, and the secular time trend are searched for in the range {1,…,20}×{1,…,10}×{1,…,5}×{1,…,20} by minimizing the AIC value. The degrees selected were 5 for temperature, 1 for humidity, 1 for temperature range, and 19 for time trend. Upon inspection of the model fit with these as the degrees of the respective polynomial effect functions, the term associated with humidity was found insignificant, and therefore was dropped from the model for further reduced AIC value and simplified model. In the simplified model the indicator variable for the SARS pandemic was also insignificant and therefore dropped. The significant terms remaining in the thus simplified best fitting Poisson GLM are the 5th degree polynomial of temperature, the 1st degree polynomial (linear) function of temperature range, the terms for weekday effects, the terms for month effects, and the 19th degree polynomial for the secular time trend.

**Fig. 5:**
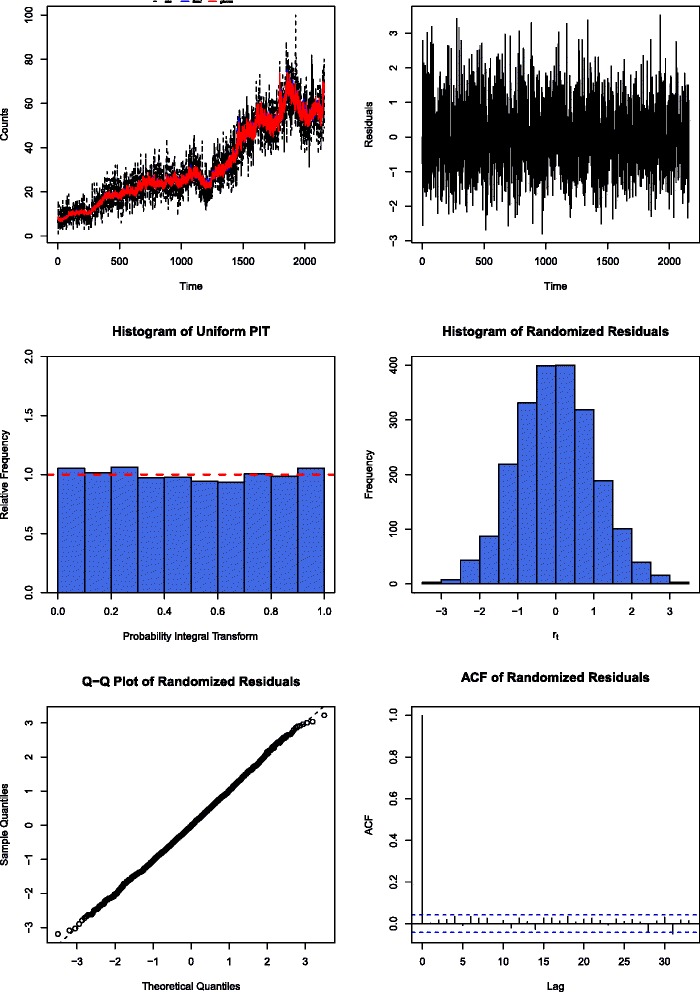
Diagnostic plots of the negative binomial GLARMA model. Top left: Observed daily call counts versus the fitted values by the GLARMA model; Top right: plot of the Pearson residuals against time; Middle left: histogram of the PIT residuals; Middle right: histogram of the normalized (randomized) PIT residuals; Bottom left: QQ plot of the randomized residuals; Bottom right: ACF plot of the randomized residuals

Inspection of the time series of the Pearson residuals from fitting the simplified model revealed significant auto-correlation at lags 1, 5, 13, 19, 23, and 33. Refitting a Poisson GLARMA model with these as the auto-regressive lags to the data left no significant auto-correlation among the residual time series. The residual deviance after fitting the Poisson GLARMA model was 2741.1 on 2109 degrees of freedom, indicating moderate overdispersion of data relative to the Poisson model. Therefore, a negative binomial GLARMA model was refitted to the data. The diagnostic plots for this model are shown in Fig. 5. It reveals fitted values for the response closely tracing the observed counts, white-noise looking residual time series that have no trend or serial correlation, and uniformly distributed PIT (Probability Integral Transform) residuals [11, 12]. These observations suggest that the final negative binomial GLARMA model produces adequate fit to the data.

**Fig. 6:**
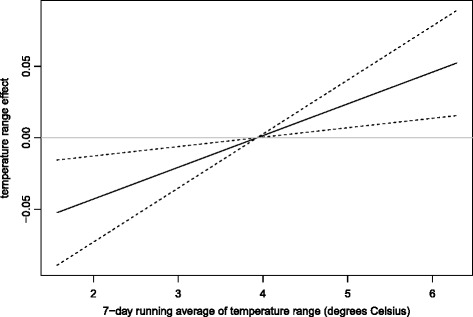
Plot of the estimated effect function of temperature range. Dashed lines indicate the 95 % confidence intervals

In the final negative binomial GLARMA model, the terms associated with temperature, temperature range, day of the week, and secular time trend remain significant, while the month of year effect was only marginally significant; see Table 1. The estimated effect of temperature range on the logarithm of the call rate turns out to be linear, with a 1 °C increase in temperature range associated with an increase in the call rate by 2.24 % (95 % CI: [0.66 *%*,3.85 *%*]); see also Fig. 6. The estimated weekday effects suggest that Monday on average sees 9.34 % (95 % CI: [5.89 %,12.91 %]) more calls per 10,000 subscribers than Sunday. This might be because on Sundays it is more likely for senior citizens living alone to be visited by family members or social workers and these visits might have delayed part of the senior citizens’ need for emergency service to the beginning of the week. At level 0.05, the month effects was non-significant (*P*-value = 0.077), which seems to suggest that any marginal month effects could be due to the difference in the weather conditions in different months.

**Table 1:** Estimated regression coefficients in the final negative binomial GLARMA model

Term	Parameter	Est.	S.E.	z-value	*P*-value
Intercept		2.442531	0.039518	61.807	< 2e–16 ***
poly(avgtem,deg = 5)					6.60e–05 ***
	1	–2.107423	0.854826	–2.465	0.013689 *
	2	0.533695	0.342106	1.560	0.118753
	3	–0.002327	0.282575	–0.008	0.993430
	4	0.544087	0.245085	2.220	0.026419 *
	5	0.316611	0.223906	1.414	0.157352
poly(avgrng,deg = 1)	1	0.703833	0.252429	2.788	0.005299 **
poly(day,degree = 19)					3.32e–124 ***
	1	8.421452	0.342929	24.557	< 2e–16 ***
	2	–1.025939	0.299326	–3.427	0.000609 ***
	3	1.081812	0.400462	2.701	0.006905 **
	4	–3.635584	0.296644	–12.256	< 2e–16 ***
	5	–1.653215	0.408414	–4.048	5.17e–05 ***
	6	1.262107	0.377475	3.344	0.000827 ***
	7	1.333170	0.330431	4.035	5.47e–05 ***
	8	–1.197338	0.470180	–2.547	0.010879 *
	9	–0.076322	0.389505	–0.196	0.844651
	10	–0.862383	0.325797	–2.647	0.008121 **
	11	1.342996	0.580335	2.314	0.020658 *
	12	1.117173	0.472052	2.367	0.017951 *
	13	–1.103724	0.291459	–3.787	0.000153 ***
	14	0.647134	0.404929	1.598	0.110012
	15	–1.066383	0.731316	–1.458	0.144794
	16	–0.825548	0.738731	–1.118	0.263771
	17	0.337155	0.771639	0.437	0.662160
	18	1.236506	0.561054	2.204	0.027531 *
	19	0.923495	0.471105	1.960	0.049964 *
weekday					2.37e–11 ***
	Monday	0.089304	0.016387	5.450	5.05e–08 ***
	Tuesday	0.021804	0.014619	1.491	0.135840
	Wednesday	0.007786	0.016910	0.460	0.645188
	Thursday	0.031717	0.016603	1.910	0.056097.
	Friday	0.010251	0.015368	0.667	0.504731
	Saturday	–0.007043	0.016094	–0.438	0.661673
month					0.077200.
	February	0.042941	0.032453	1.323	0.185784
	March	0.014045	0.045926	0.306	0.759750
	April	–0.053393	0.059269	–0.901	0.367666
	May	–0.065915	0.066509	–0.991	0.321651
	June	–0.117673	0.068948	–1.707	0.087881.
	July	–0.072637	0.067278	–1.080	0.280290
	August	–0.050010	0.065466	–0.764	0.444926
	September	–0.040152	0.063196	–0.635	0.525196
	October	–0.053443	0.057428	–0.931	0.352061
	November	–0.029786	0.049083	–0.607	0.543961
	December	–0.013625	0.033611	–0.405	0.685201

**Fig. 7:**
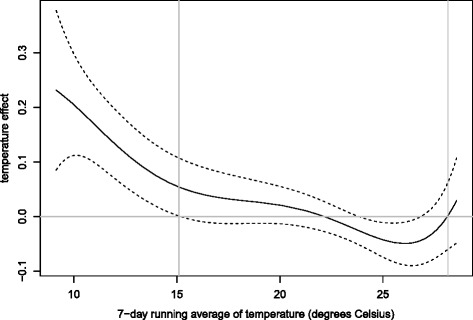
Plot of the estimated effect function of temperature. Dashed lines indicate the 95 % confidence intervals

The effect function for temperature is shown in Fig. 7, from which it is noted that the effect is highly non-linear with a U-shape roughly. Due to the nonlinearity of the effect function, the effect of one unit change in the temperature is not a constant. Here we report the average effect in different temperature intervals. When the temperature is in the range 15–24 °C, on average there is no significant change in call rate associated with the change in temperature (95 % CI [-0.25 %,2.15 %]). When the temperature is below 15 °C, a unit decrease in temperature is associated with an increase in the call rate by 3.03 % (95 % CI: [0.75 %,5.36 %]) on average. There was about 11.3 % of the days in which the temperature was 15 °C or below. When the temperature is around 28 °C or higher, increases (rather than decreases) in temperature seem to be linked with increases in the call rate, although the effect is only marginally significant.

## Discussion and conclusions

We have made an attempt to model the time series of the daily numbers of PE Link calls that lead to hospital admissions in Hong Kong. A negative binomial generalized linear auto-regressive moving-average model was found to afford adequate fit to the data. Our analysis reveals that temperature is a significant predictor for call numbers. We found the effect of the average temperature to be highly nonlinear and roughly U-shaped. The finding aligns wells with previous researches about the effect of weather on various measures of health status. By inspecting the effect function of temperature, we were able to identify a cold temperature threshold of about 15 °C, which triggers excessive PE Link calls. Our findings are potentially useful in assisting the SCHSA with service planning and resource allocation.
